# Status and Factors of Cognitive Function Among Older Adults in Urban China

**DOI:** 10.3389/fpsyg.2021.728165

**Published:** 2021-09-08

**Authors:** Lei Shen, Xiaochen Tang, Chunbo Li, Zhenying Qian, Jijun Wang, Wei Liu

**Affiliations:** ^1^Department of Psychology, Shanghai Normal University, Shanghai, China; ^2^Shanghai Key Laboratory of Psychotic Disorders, Shanghai Mental Health Center, Shanghai Jiao Tong University School of Medicine, Shanghai, China

**Keywords:** cognitive function, influencing factors, older adults, urban community, healthy aging

## Abstract

The present study aimed to examine the current status and influencing mechanisms of different demographic factors associated with cognitive function in urban Chinese older adults. A total of 644 older adults from 14 communities in urban China (e.g., Shanghai, Beijing, and Wuxi) were investigated by using the Mini-Mental State Examination and the Repeatable Battery for the Assessment of Neuropsychological Status. The results indicated that the overall cognitive function of older adults in urban China was normal. We found an aging effect on cognitive level, and cognitive function declined more rapidly after age 80. Older age, unmarried status, and lower occupational cognitive requirements increased the likelihood of cognitive risk. Higher educational levels and active engagement in exercise may contribute to cognitive reserve and have a protective effect on cognitive decline in late life. Further study is needed to develop appropriate interventions to improve the mental health of older people.

## Introduction

Aging has become an important public health issue due to the acceleration of the global population aging process, particularly in developing countries. China is an aging society with the largest older population in the world (Han et al., 2020). The number of older people has increased rapidly at an unprecedented rate in the past few decades. By the end of 2019, the number of people aged 60 and older reached approximately 254 million, accounting for 18.1% of the total population in China (National Bureau of Statistics of China, 2020). It is estimated that the older population will account for more than 30% in 2050, showing a continuously increasing aging trend. The rapid aging situation brings major challenges to the healthy development and security of the older people in China.

Cognitive maintenance is an important aspect of healthy aging (Zaninotto et al., 2018). Cognitive function includes attention, memory, language, processing speed, visuospatial ability, and executive function (Harada et al., 2013). Accumulating evidence has demonstrated that cognitive function progressively decline with age (Legdeur et al., 2017; Cornelis et al., 2020), primarily due to the structural and functional changes in aging brains (Murman, 2015). Many studies have shown that cognitive aging leads to an increased risk of cognitive impairment and directly affects an individual’s physical and mental condition, further decreasing the quality of life (Bettio et al., 2017; Liang et al., 2019). As population aging is becoming more severe in urban China, the problem of cognitive aging accompanied by rapid older population growth is more prominent (Xiang et al., 2018), which has drawn great attention of researchers.

Given that cognitive function is an important basis for mental health (Kim et al., 2017) and a key indicator of aging (Hou et al., 2020), how to delay cognitive decline has become a focus in public health research (Yu et al., 2021). The cognitive reserve hypothesis emphasizes the role of experience in the active model of cognitive reserve (Stern, 2012). It argues that educational level, career achievement, and social activities are closely related to the level of cognitive function. These factors can foster dynamic changes in cognitive development and affect the underlying mechanisms of brain function, which contribute to delayed cognitive aging (Stern et al., 2020). Some studies have identified the influencing factors associated with cognitive function in older adults. Previous research has shown that higher educational level and active social engagement are protective factors for cognitive function (Clouston et al., 2019; Dause and Kirby, 2019), which can regulate the aging effect on cognitive performance and slow down cognitive decline caused by aging to some extent to reduce the risk of cognitive impairment (Opdebeeck et al., 2016). Furthermore, regular exercise has effective impact on preventing cognitive decline (Barnes, 2015; Northey et al., 2018). Hu et al. (2014) developed a 6-month exercise plan for 96 older adults with mild cognitive impairment and observed that the exercise program significantly improved cognitive function after the intervention.

Evidence from the developed countries has demonstrated that cognitive function is affected by multiple factors, while the underlying mechanism of the above influencing factors on cognitive function of older adults in urban China remains unclear. To date, most studies have focused on the assessment of cognitive impairment in the older adults (Yu et al., 2012; Hu et al., 2013) and a few studies have examined the influence of some factors on cognitive function in urban Chinese older adults (Ren et al., 2018; Han et al., 2019). However, these studies discussed the overall level of cognition, largely ignoring the specific cognitive domains. Moreover, the relatively limited sample coverage of previous studies is also insufficient to elucidate the mechanisms of multiple factors on cognitive function (Zhang et al., 2019). Considering the different cognitive function patterns for individuals at different age stages (Matthews et al., 2013), it will have potential implications to further reveal the status of cognitive development and the mechanisms of related influencing factors on cognitive function to gain a more comprehensive understanding of the global cognition and various cognitive domains in urban Chinese older adults at different ages.

In this study, we aimed to explore the current status of cognitive function in urban Chinese older adults and the impact of demographic factors (including gender, age, educational level, marital status, occupational status, and regular exercise) on different cognitive domains. In order to comprehensively assess the cognitive level of older people, we used the Mini-Mental State Examination (MMSE) and Repeatable Battery for the Assessment of Neuropsychological Status (RBANS), which have been verified to have reasonable sensitivity and specificity for cognitive impairment in previous studies (Cheng et al., 2011; Suraweera et al., 2016). The findings of the current study are expected to gain better insights into how to reduce cognitive risk and promote healthy aging.

## Materials and Methods

### Participants

According to the age classification criteria in China, citizens aged 60years and above were identified as the senior citizens. In the present study, we recruited 814 older adults from 14 communities in Shanghai, Beijing, Wuxi, and other cities nationwide. About 644 (79.1%) valid data were retained for the study, including 231 males and 413 females aged 60–99years (*M*=72.97; *SD*=7.64). The inclusion criteria included: (1) aged 60years or older; (2) had no disability, and no difficulties in hearing, vision, or communication; and (3) had no severe mental illnesses. The exclusion criteria included: (1) prior diagnoses of psychiatric disorders (e.g., depressive disorder and schizophrenia); (2) major physical illness; and (3) history of alcohol and drug dependence. The study was followed the principles of the ethical committee of Shanghai Mental Health Center and the Declaration of Helsinki of 1964. All the participants signed a written informed consent before answering the questionnaires.

### Measuring Instruments

#### Demographic Characteristics

The general information questionnaire was used to obtain demographic information from participants, including six items: gender, age, educational level, marital status, occupational status, and regular exercise.

#### Cognitive Function

The MMSE is the most commonly used tool developed by Folstein et al. (1975) for assessing global cognition and screening cognitive impairment in the older population. A modified Chinese version of the MMSE was used in this study (Katzman et al., 1988). It consists of 19 items with a total score of 30, covering multiple cognitive domains including visual space, memory, attention, calculation, abstraction, orientation, and verbal ability. According to the previous studies (Zhou et al., 2006; Cui et al., 2011), the education-based MMSE scores defined the cut-off points of cognitive impairment as following: ≤17 for the illiterate group (no formal education), ≤20 for the group with primary education (education ≤6years), and ≤24 for the group with middle education and above (education >6years). The reliability and validity of the Chinese version of the MMSE have been validated in Chinese older adults (Zhang et al., 1990). In this study, the Cronbach’s alphas value of the MMSE was 0.80.

The RBANS is a widely used neuropsychological screening tool developed by Randolph et al. (1998). It comprised 12 subtests to comprehensively evaluate five cognitive domains across a wider age range. The RBANS has been demonstrated to be valid and reliable for detecting cognitive impairment in various populations (Duff et al., 2008; McKay et al., 2008). A modified Chinese version of the RBANS was used in this study (Cheng et al., 2011). The RBANS consists of the immediate memory (including the list learning and story memory subtests), the visuospatial/constructional (including the figure copy and line orientation subtests), the language (including picture naming and semantic fluency subtests), the attention (including the digit span and coding subtests), and the delayed memory (including the list recall, list recognition, story recall, and figure recall subtests). The raw scores were converted to scaled scores with a mean of 100 and a SD of 15. The Chinese version of the RBANS demonstrated satisfactory validity and reliability for the healthy older people in China (Cheng et al., 2011; Jiang et al., 2014). The Cronbach’s alphas value of the RBANS total scale was 0.93 in this study.

### Research Procedure

In the present study, we adopted a questionnaire method. Before the survey, all staff members received unified training. The survey was conducted face-to-face. For the participants who understood the meaning of the questions unclearly or who had a low educational level, the experimenter explained each item individually and assisted with reading until the items were fully understood.

### Statistical Analysis

All data were statistically analyzed using IBM SPSS Statistics 25.0 (IBM SPSS Corp, Armonk, NY, United States). Descriptive statistics were used to analyze the characteristics of the participants. Independent samples *t*-test and one-way ANOVA were used to compare the cognitive function of urban Chinese older adults under different socio-demographic factors. Binary logistic regression analysis and multivariate linear regression analysis were, respectively, used to examine the predictors associated with cognitive impairment and cognitive function. Multivariate regression models were established with cognitive impairment (with cognitive impairment=1, without cognitive impairment=0) and the MMSE and RBANS scores as dependent variables. The independent variables were identified as statistically significance in the univariate analyses (coding marital status, occupational status, and regular exercise as dummy variables) and entered into the regression models using a stepwise method. The results of binary logistic regression analysis were presented as odds ratios (OR) with a 95% CI. All results were considered statistically significant when *p*<0.05.

## Results

### The Current Cognitive Status Among Older Adults in Urban China

Descriptive statistical analysis shown in Table 1 indicated that the mean MMSE total score was 26.80 (3.89). According to the modified Chinese version of the MMSE, we identified 563 older adult individuals with normal cognitive function and 81 older adult individuals with cognitive impairment (12.6%). The mean RBANS total score was 87.04 (16.73). The mean and *SD* of the RBANS index scores were presented in Table 1.

**Table 1:** Cognitive status among older adults in urban China (*N*=644).

*CF*	*M*	*SD*
MMSE	26.80	3.89
RBANS	87.04	16.73
RBANS index	*M*	*SD*
Immediate memory	78.32	17.56
Visuospatial/constructional	93.73	18.15
Language	94.40	12.99
Attention	96.76	18.38
Delayed memory	87.89	20.00

### Univariate Analyses of Factors Related to Cognitive Function Among Older Adults in Urban China

We used univariate analysis with the MMSE and RBANS total scores to examine the influence of factors (gender, age, educational level, marital status, occupational status, and regular exercise) on cognitive function. As shown in Table 2, the MMSE total scores were significantly different in age (*p*<0.001), educational level (*p*<0.001), marital status (*p*<0.01), occupational status (*p*<0.01), and regular exercise (*p*<0.05). The RBANS total scores were significantly different in age (*p*<0.001), educational level (*p*<0.001), occupational status (*p*<0.001), and regular exercise (*p*<0.05).

**Table 2:** Univariate analysis for factors of cognitive function among older adults in urban China.

Variable	Group	*N*	MMSE	*t/F*	*d*/*η*^2^	RBANS	*t/F*	*d*/*η*^2^
Gender	Male	231	26.68 (4.17)	−0.59	−0.04	86.82 (15.72)	−0.25	−0.02
	Female	413	26.87 (3.73)			87.16 (17.28)		
Age (years)	60–69	243	27.79 (0.23)	43.63^***^	0.17	89.12 (13.41)	35.74^***^	0.14
	70–79	258	27.38 (0.22)			91.31 (15.54)		
	80–89	132	24.49 (0.31)			76.75 (18.83)		
	90–99	11	19.27 (1.07)			63.36 (14.37)		
Educational level	Illiteracy	29	20.28 (3.94)	32.54^***^	0.20	57.04 (10.43)	58.81^***^	0.32
	Primary	44	23.57 (5.75)			69.34 (18.05)		
	Middle	207	26.87 (4.17)			84.47 (13.69)		
	Senior	213	27.62 (2.60)			90.31 (13.25)		
	Undergraduate	147	27.75 (2.43)			96.55 (14.14)		
	Postgraduate	4	28.25 (2.36)			101.00 (17.38)		
Marital status	Married	500	27.06 (3.45)	3.18^**^	0.02	87.86 (16.14)	1.67	0.01
	Widowed	119	26.04 (5.20)			85.12 (18.27)		
	Divorced	15	25.73 (3.33)			79.60 (18.66)		
	Separated	2	27.00 (4.24)			87.50 (7.78)		
	Never married	5	22.00 (6.89)			76.20 (22.40)		
	Other	3	27.67 (3.22)			80.33 (25.01)		
Occupational status	Public institution	121	27.28 (2.98)	3.72^**^	0.04	91.31 (15.56)	8.15^***^	0.08
	Technician	149	27.28 (3.32)			90.64 (15.53)		
	Clerk	96	27.71 (2.99)			91.37 (13.81)		
	Commercial service	75	25.37 (5.72)			82.20 (19.37)		
	Husbandry production	8	25.88 (3.27)			79.88 (20.03)		
	Equipment operation	142	26.23 (4.27)			80.98 (16.50)		
	Solider	4	29.00 (0.82)			97.00 (11.46)		
	Other	49	26.24 (3.91)			82.27 (16.36)		
Regular exercise	Yes	416	27.04 (3.70)	1.97^*^	0.17	88.19 (16.30)	2.36^*^	0.19
	No	228	26.38 (4.20)			84.94 (17.33)		

* *p<0.05*;

** *p<0.01*;

*and*

*** *p<0.001*.

From the results of *post hoc t*-test comparisons, we identified that the MMSE and RBANS in the older adults aged 60–69 and 70–79 scored significantly higher than those aged 80–89 (*p*<0.001); all scored significantly higher than those aged 90–99 (*p*<0.01). Moreover, older adults without any formal education or with primary education had lower MMSE and RBANS scores (*p*<0.01). The MMSE and RBANS total scores in the never-married older adults were significantly lower than those in the married, divorced, and widowed older adults (*p*<0.05). Regarding the influence of occupational status on MMSE and RBANS, the differences were mainly among the commercial service staff, public institution staff, technicians, and clerks. The former scored significantly lower than the latter three (*p*<0.01).

### Regression Analyses of Factors Related to Cognitive Function Among Older Adults in Urban China

According to the classification criteria for cognitive impairment in the MMSE, binary logistic regression analysis was conducted with statistically significant variables in univariate analyses as independent variables. Table 3 indicated that age (OR=1.12, 95% CI=1.08–1.15) and educational level (OR=0.72, 95% CI=0.56–0.93) were significant predictors of cognitive impairment. Based on the results of multivariate linear regression analysis with the MMSE and RBANS total scores as dependent variables, Table 4 indicated that age and educational level both had significant effects on the MMSE and RBANS (*p*<0.001). The married and widowed older adults scored higher on the MMSE than the never-married older adults (*p*<0.01).

**Table 3:** Binary logistic regression of the association between cognitive impairment and factors.

Independent variable	*B*	OR	95% CI	*p*
Age (years)	0.11	1.12	1.08–1.15	<0.001
Educational level	−0.33	0.72	0.56–0.93	0.01
Regular exercise (reference: no)	−0.27	0.76	0.45–1.28	0.30
**Marital status (reference: never married)**				
Married	−1.83	0.16	0.02–1.12	0.07
Widowed	−2.35	0.10	0.01–0.74	0.02
Divorced	−0.20	0.82	0.08–8.10	0.86
Separated	0.78	2.18	0.07–71.13	0.66
Other	−21.61	0.00	0.00	1.00
**Occupational status (reference: commercial service)**				
Public institution	−0.45	0.64	0.26–1.58	0.33
Technician	−0.18	0.84	0.37–1.93	0.68
Clerk	−0.29	0.75	0.29–1.96	0.56
Husbandry production	0.57	1.77	0.28–11.03	0.54
Equipment operation	−0.54	0.58	0.26–1.32	0.20
Solider	−19.34	0.00	0.00	1.00
Other	−0.84	0.43	0.13–1.50	0.19

**Table 4:** Multivariate linear regression of factors related to the Mini-Mental State Examination (MMSE) and Repeatable Battery for the Assessment of Neuropsychological Status (RBANS).

Independent variable	MMSE	RBANS
	*B* (*SE*)	*B* (*SE*)
Age (years)	−0.18 (0.02)^***^	−0.59 (0.07)^***^
Educational level	1.21 (0.14)^***^	7.67 (0.57)^***^
Regular exercise (reference: no)	0.24 (0.28)	1.03 (1.12)
**Marital status (reference: never married)**		
Married	5.10 (1.51)^**^	12.80 (6.09)
Widowed	5.11 (1.54)^**^	14.35 (6.19)
Divorced	3.30 (1.74)	3.56 (7.02)
Separated	3.97 (2.80)	5.21 (11.27)
Other	7.19 (2.45)^**^	13.29 (9.88)
**Occupational status (reference: commercial service)**		
Public institution	0.59 (0.50)	1.96 (2.03)
Technician	0.61 (0.49)	1.07 (1.98)
Clerk	0.89 (0.52)	2.06 (2.11)
Husbandry production	0.03 (1.24)	−3.96 (4.99)
Equipment operation	0.72 (0.48)	−0.95 (1.93)
Solider	2.62 (1.71)	9.84 (6.90)
Other	0.64 (0.61)	−0.03 (2.47)
*F*	16.73^***^	24.91^***^
Adjusted *R*^2^	0.27	0.36

^*^p<0.05;

** *p<0.01*;

*and*

*** *p<0.001*.

We used multivariate linear regression analysis to examine the association between the RBANS index and the above factors. As presented in Table 5, age negatively predicted the RBANS index (*p*<0.001), while educational level positively predicted the RBANS index (*p*<0.001). Moreover, regular exercise and marital status were the significant predictors of the RBANS index. Older adults who regularly participate in exercise showed higher scores for the immediate memory (*p*<0.01). The married and widowed older adults showed higher attention scores compared to the never-married older adults (*p*<0.05), and the widowed older adults scored higher on the delayed memory than the never-married older adults (*p*<0.01).

**Table 5:** Multivariate linear regression of the association between the RBANS index and factors.

Independent variable	Immediate memory	Visuospatial/ constructional	Language	Attention	Delayed memory
	*B* (*SE*)	*B* (*SE*)	*B* (*SE*)	*B* (*SE*)	*B* (*SE*)
Age (years)	−0.31 (0.08)^***^	−0.42 (0.09)^***^	−0.31 (0.06)^***^	−0.65 (0.08)^***^	−0.82 (0.09)^***^
Educational level	6.92 (0.66)^***^	5.46 (0.71)^***^	4.05 (0.51)^***^	8.25 (0.64)^***^	7.25 (0.72)^***^
Regular exercise (reference: no)	3.84 (1.29)^**^	0.13 (1.39)	1.07 (1.00)	−0.99 (1.26)	1.40 (1.42)
**Marital status (reference: never married)**					
Married	7.21 (6.98)	13.24 (7.52)	7.10 (5.41)	17.22 (6.83)^*^	14.20 (7.71)
Widowed	9.74 (7.10)	14.55 (7.65)	6.27 (5.50)	17.79 (6.96)^*^	16.40 (7.84)^**^
Divorced	1.99 (8.06)	4.55 (8.68)	−0.03 (6.24)	6.78 (7.89)	3.79 (8.90)
Separated	−13.64 (12.93)	17.43 (13.92)	6.67 (10.02)	9.44 (12.66)	9.42 (14.28)
Other	3.22 (11.33)	5.85 (12.20)	6.87 (8.78)	24.57 (11.10)^*^	22.31 (12.52)
**Occupational status (reference: commercial service)**					
Public institution	1.44 (2.33)	1.75 (2.51)	2.59 (1.81)	1.44 (2.28)	1.79 (2.57)
Technician	0.06 (2.27)	3.14 (2.45)	0.97 (1.76)	0.23 (2.23)	−0.49 (2.51)
Clerk	3.52 (2.42)	0.97 (2.60)	1.61 (1.87)	−1.62 (2.37)	4.24 (2.67)
Husbandry production	−2.59 (5.72)	−2.90 (6.16)	−0.66 (4.43)	−0.66 (5.60)	−6.99 (6.32)
Equipment operation	−0.57 (2.21)	−2.26 (2.38)	0.91 (1.71)	−1.07 (2.17)	−0.19 (2.44)
Solider	14.04 (7.92)	2.76 (8.53)	2.23 (6.13)	5.55 (7.75)	14.50 (8.75)
Other	0.33 (2.84)	−0.16 (3.06)	2.67 (2.20)	−2.69 (2.78)	0.04 (3.13)
*F*	14.10^***^	9.72^***^	9.18^***^	22.06^***^	17.62^***^
Adjusted *R*^2^	0.23	0.17	0.16	0.33	0.28

* *p<0.05*;

** *p<0.01*;

*and*

*** *p<0.001*.

## Discussion

In a larger sample of urban Chinese older adults aged 60 and above, the present study assessed the current status of cognitive function and explored the influence of different demographic factors on cognitive function. It turned out that cognitive function of older people in urban China was normal. The current study comprehensively demonstrated the combined contribution of multiple demographic factors to cognitive function. There was an aging effect on cognitive level, and cognitive function declined more rapidly after age 80. From the results of binary logistic regression analysis and multivariate linear regression analysis, we found age, educational level, marital status, occupational status, and regular exercise were the significant predictors of cognitive function. Older age, unmarried status, and lower occupational cognitive requirements were the risk factors of cognitive function. Higher educational levels and regular exercise were protective factors that contribute to cognitive maintenance and enhancement. The findings provide new empirical evidence and directions for preventing cognitive aging and promoting healthy aging.

This study investigated the cognitive level in urban Chinese older people showing that the mean MMSE total score of older adults was 26.80, which was higher than the education-based MMSE cut-off points for older adults with senior education and above. The mean RBANS total score was 87.04 (16.73), which was significantly lower than the mean of the American norm 100 (15; Randolph et al., 1998). Furthermore, the mean RBANS total scores and RBANS index scores in the current study were lower than those in studies of healthy older adults in developed countries (Karantzoulis et al., 2013; Hammers et al., 2020). This might partially result from the influence of educational level and cultural differences. However, the mean RBANS total score in this study was similar to the results of previous surveys of healthy older adults from Beijing (Zhang et al., 2008), Shanghai (Cheng et al., 2011), and different regions in China (Jia et al., 2019), indicating that the overall cognitive function level of older adults in urban China was normal. It may imply that as urban living standards, lifestyles, and educational level have greatly changed and improved, older adults in urban areas experience less cognitive decline (Xue et al., 2018). Nevertheless, evidence from a recent research showed that cognitive decline rate was faster in urban older adults, which largely affected by some risk factors in urban settings, such as high population density and healthcare services (Xiang et al., 2018). More research should further investigate other factors linked to cognitive decline and compare the cognitive level of older population in urban and rural China.

We conducted univariate analysis to examine the demographic characteristics of cognitive function and observed an aging effect on the cognitive function of older adults in urban China. Aging was a risk factor leading to cognitive deficits and significantly negatively predicted cognitive function. These results confirmed the previous conclusion of a declining trend in cognitive function with aging (Zamora-Macorra et al., 2017). Normal aging is accompanied by changes in cognitive function and brain function. Studies have confirmed that age is closely related to cognitive function, and cognitive decline is an obvious feature of aging (Brewster et al., 2014). Our study also expanded the previous findings by examining the cognitive levels at different age stages. Combined with the MMSE, RBANS total scores, and the RBANS index scores, we found that the age of 80 was a key age for the rapid decline of cognitive function in urban Chinese older adults. Further analysis revealed that the MMSE and RBANS total scores in individuals aged 80–89 were significantly higher than those in individuals aged 90 and above. Specifically, the attention, the immediate memory, and the delayed memory scores were found to be better. However, the results were inconsistent with a previous study that investigated the cognitive pattern of more than 3,000 community-dwelling American residents aged 45–99 (Hoogendam et al., 2014). They found that MMSE remained stable until the age of 70 and then showed a significant declining trend, while age had no strong effect on MMSE. In the related cognitive indices, fine motor skills, processing speed, and visuospatial ability were significantly affected, while verbal fluency and memory were less affected. In contrast, age was a significant negative predictor of MMSE in the current study and had a strong effect on the attention and delayed memory. This may be due to the differences of selected respondents. In the current study, the respondents were mainly aged 60 and above, and 12.6% of older adults with cognitive impairment were included, while the previous study recruited healthy middle-aged and older adults. Furthermore, the aging effect on specific cognitive dimensions was different, which could be due to the difference in the cognitive screening tools. Further age division is needed to clarify the cognitive change in older adults with more comprehensive measurements.

This study also demonstrated that marital and occupational status were the important factors affecting the cognitive function of older adults in urban China. The overall cognitive level of older adults who had never married was significantly lower than that of married, divorced, or widowed older people. The result was similar to the previous studies on older adults in urban areas in China and developed countries (Feng et al., 2014; Sommerlad et al., 2018). Based on the findings of a large-scale investigation of the impact of marital status on cognitive impairment in Chinese community-dwelling older people, never-married older adults may experience higher cognitive risk, with a rate of cognitive impairment about 2.5 times higher than that of older adults with other marital status (Feng et al., 2014). Lifelong single individuals are those who live alone and lack the necessary emotional support, thus more negative emotions, such as loneliness and depression, may be elicited. Unmarried individuals also showed lower social engagement, as well as the availability of sociopsychological resources, which could affect cognitive health and constitute a risk factor for cognitive decline (Sommerlad et al., 2018). However, a recent investigation of older Americans revealed that divorced and widowed status had a significant negative impact on cognitive function (Lam et al., 2019). This result was not reflected in our study and one explanation for this discrepancy was that the effect of marital status on cognitive function might be moderated by other factors (Xu et al., 2021). Therefore, the association between marital status and cognitive function in older people should be further explored. In terms of occupational status, we found that the older people engaged in public institutions, technicians, and clerical work had a higher cognitive level, while the cognitive level was lower in older adults who had previously engaged in commercial services as their main occupation, indicating that lower occupational cognitive requirements may increase the risk of cognitive decline. This finding was supported by the previous studies. A longitudinal study examined the relationship between occupational cognitive requirements and rates of cognitive decline (Pool et al., 2016). They found that occupational cognitive requirements had a significant positive effect on cognitive performance in old age. Similarly, Andel et al. (2016) examined the effects of preretirement work complexity on cognitive function and revealed that even after retirement, older adults exposed to more complex occupations still had higher levels in cognitive domains such as memory, language, and processing speed. Various occupational activities reflect differences in cognitive requirements, and the complexity of occupational characteristics determines the level of cognitive reserve (Chapko et al., 2018). Therefore, older adults who have been engaged in higher occupational cognitive requirements for a long time can maintain better cognitive function.

Another important finding in this study was that educational level and regular exercise were significant positive predictors of cognitive function in urban older adults. We found that the cognitive function of older adults with senior education and above was better on the MMSE and RBANS total scores, indicating that a higher educational level had a protective effect on cognitive function and could reduce cognitive risk. This result validated the cognitive reserve hypothesis and was consistent with the results of previous studies (Meng and D’Arcy, 2012; Sattler et al., 2012). The cognitive reserve hypothesis points out that educational level is an important factor in improving cognitive reserve and plays a positive compensatory role in the cognitive aging process (Stern, 2012). A longitudinal study found that older individuals with lower educational levels experienced a long period of sustained cognitive decline before Alzheimer’s dementia (Amieva et al., 2014). The findings were also supported by the indirect evidence from neuroimaging studies that educational level was positively associated with gray matter and white matter volume (Foubert-Samier et al., 2012). It suggested that individuals with higher education were able to acquire a higher level of cognitive reserve to cope with brain changes related to cognitive aging and thus maintain a higher cognitive level. From the perspective of social development, education has a significant effect on individual development. Individuals with higher education have advantages in some social factors (e.g., social resources, social status, and career achievement) that have a promoting effect on cognitive function (Lövdén et al., 2020). In the current study, we also observed that older adults who exercised regularly had higher MMSE and RBANS scores, suggesting that engaging in exercise frequently could improve overall cognitive function. This confirmed the previous studies among older urban adults in developed countries (Gothe, 2018; Kramer and Colcombe, 2018). From the physiological and neurological level, regular exercise helps maintain the integrity of the cardiovascular system, promote blood circulation, and reduce the risk of neurodegenerative diseases (Barnes, 2015). At the psychological and behavioral level, regular participation in exercise indicates the possession of healthy lifestyle and positive attitude toward life (Clare et al., 2017). The maintenance of positive emotions and behaviors contributes to physical and mental health development (Livingston et al., 2017). It was worth noting that the current study revealed that the effect of exercise on cognitive function in urban Chinese older adults manifested in the immediate memory index. This finding indicated that regular exercise could promote memory performance. One explanation for the result was that memory enhancement might result from improvements in cognitive and neural reserve through exercise. An fMRI study of older individuals with mild cognitive impairment revealed that activation of brain areas associated with semantic memory retrieval was significantly reduced and showed increased activation in the frontal lobe after the 12-week exercise intervention, suggesting that active engagement in exercise improved neural efficiency and cognitive function (Smith et al., 2013). These findings in the present study will contribute to the development of interventional strategies for cognitive aging and promote long-term cognitive effects.

There were several limitations of the study should be mentioned. Firstly, this study was a cross-sectional study that understood the association between the demographic factors and cognitive function was limited, and the causal relationship could not be inferred. Therefore, it is necessary to conduct a large-scale longitudinal study of cognitive trajectories in older adults. Secondly, the current study only investigated the older adults in urban China, and the conclusions obtained from the study were not generalizable. Future research should further compare the cognitive function and the relationship between related influencing factors and cognitive aging in rural and urban Chinese older people. In addition, this study explored the effects of different demographic factors on cognitive function, while the factors affecting cognitive aging were not limited in the present study. It should take more various psychological and environmental variables into consideration to further analyze the interactions and specific mechanisms of different factors on cognitive function.

## Conclusion

The present study found that the overall cognitive function in urban Chinese older adults was normal and was greatly affected by age, educational level, marital status, occupational status, and exercise. There was an obvious aging effect on cognitive level, and cognitive function declined more rapidly after age 80. Cognitive function in the never-married older adults was lower. Older individuals used to be engaged in higher occupational cognitive requirements showed better cognitive levels. The protective effect of higher educational level and regular exercise on cognitive function could develop appropriate interventions to prevent and reduce cognitive aging.

## Data Availability Statement

The raw data supporting the conclusions of this article will be made available by the authors, without undue reservation.

## Ethics Statement

The studies involving human participants were reviewed and approved by the ethical committee of Shanghai Mental Health Center. The patients/participants provided their written informed consent to participate in this study.

## Author Contributions

JW and WL contributed to the conceptualization of the study. LS and XT contributed to perform the statistical analysis and write the manuscript. CL and ZQ contributed to the data collection. All authors contributed to manuscript revision and approved the submitted version.

## Funding

This study was supported by the Shanghai Planning Project of Philosophy and Social Science (2017BSH009).

## Conflict of Interest

The authors declare that the research was conducted in the absence of any commercial or financial relationships that could be construed as a potential conflict of interest.

## Publisher’s Note

All claims expressed in this article are solely those of the authors and do not necessarily represent those of their affiliated organizations, or those of the publisher, the editors and the reviewers. Any product that may be evaluated in this article, or claim that may be made by its manufacturer, is not guaranteed or endorsed by the publisher.
